# Concept and considerations of a medical device: the active noise cancelling incubator

**DOI:** 10.3389/fped.2023.1187815

**Published:** 2023-07-03

**Authors:** Artur C. Jaschke, Arend F. Bos

**Affiliations:** ^1^ Department of Paediatrics, Division of Neonatology, Beatrix Children’s Hospital, University Medical Center Groningen, University of Groningen, Groningen, Netherlands; ^2^ Department of Music Therapy, ArtEZ University of the Arts, Enschede, Netherlands; ^3^ Cambridge Institute for Music Therapy Research, Anglia Ruskin University, Cambridge, United Kingdom

**Keywords:** NICU (neonatal intensive care unit), active noise cancellation (ANC), artificial intelligence, machine learning, ducted system, music-based therapy, neural development

## Abstract

**Background:**

An increasingly 24/7 connected and urbanised world has created a silent pandemic of noise-induced hearing loss. Ensuring survival to children born (extremely) preterm is crucial. The incubator is a closed medical device, modifying the internal climate, and thus providing an environment for the child, as safe, warm, and comfortable as possible. While sound outside the incubator is managed and has decreased over the years, managing the noise inside the incubator is still a challenge.

**Method:**

Using active noise cancelling in an incubator will eliminate unwanted sounds (i.e., from the respirator and heating) inside the incubator, and by adding sophisticated algorithms, normal human speech, neonatal intensive care unit music-based therapeutic interventions, and natural sounds will be sustained for the child in the pod. Applying different methods such as active noise cancelling, motion capture, sonological engineering. and sophisticated machine learning algorithms will be implemented in the development of the incubator.

**Projected Results:**

A controlled and active sound environment in and around the incubator can in turn promote the wellbeing, neural development, and speech development of the child and minimise distress caused by unwanted noises. While developing the hardware and software pose individual challenges, it is about the system design and aspects contributing to it. On the one hand, it is crucial to measure the auditory range and frequencies in the incubator, as well as the predictable sounds that will have to be played back into the environment. On the other, there are many technical issues that have to be addressed when it comes to algorithms, datasets, delay, microphone technology, transducers, convergence, tracking, impulse control and noise rejection, noise mitigation stability, detection, polarity, and performance.

**Conclusion:**

Solving a complex problem like this, however, requires a de-disciplinary approach, where each discipline will realise its own shortcomings and boundaries, and in turn will allow for innovations and new avenues. Technical developments used for building the active noise cancellation-incubator have the potential to contribute to improved care solutions for patients, both infants and adults.

Code available at: 10.3389/fped.2023.1187815.

## Introduction

Worldwide, preterm birth is the main cause of death for children under the age of 5 and was responsible for approximately 1 million deaths in 2015 (1, 2). Especially, extremely preterm born children, i.e., before 28 weeks of gestation, have a low survival rate. Upon birth, these infants are treated at neonatal intensive care units (NICUs), where incubators support their underdeveloped organs as much as possible. However, despite this highly specialised care, still approximately 40% of the extremely preterm but viable infants at 24–25 weeks will not survive. Of all infants that do survive, another ∼60% will suffer life-long health complications, including developmental delay and mild to moderate motor, cognitive, and behavioural problems (1–3). Every extra day an infant can spend in the safe uterine environment directly correlates to its chances of survival and living a high-quality life (1, 2).

The work of clinicians around preterm births is frustrating: little can be done to delay birth or to improve outcomes. Hence, prevention of the high mortality rate and severe health complications in extremely preterm infants requires a breakthrough innovation. Besides the several morbidities, a child faces in the very early stages after birth, one major and often overlooked aspect is the overstimulation through noise.

The human auditory system has its own developmental cycle with anatomical parts like the cochlea in the middle ear being well formed by 15 weeks’ gestational age (3). At 20 weeks, this structure is anatomically functional and the infant will need auditory experiences like language, music, and meaningful environmental sounds during the last 10–12 weeks of foetal life (3–5). At 25–29 weeks, the auditory system becomes functional connecting inner hair cells of the cochlea to the brain stem, thalamus, and temporal lobe. Loud and prolonged noises affect the infants’ hearing through changes in autonomic functions and impact heart rate, blood pressure, respiratory patterns, gastrointestinal motility, and, in turn, neural connectivity (3–5).

Preterm babies are constantly exposed to noise, among others, from the monitors, oxygen supply, medical devices, and general sounds on the unit. Although there are regulations and guidelines to minimise the noise on the ward, suggesting noise levels of 20–35 dB (6), these levels are often exceeded (6–14). Listening and hearing varies strongly between an intrauterine and an extrauterine exposure, especially in the NICU, and thus in an incubator environment. Mainly low frequencies, particularly those below 500 Hz, are perceived and transmitted through the mother's womb, while the NICU is often flooded with high frequency sounds, unfamiliar *hissing* and *sissing* sounds in the incubator itself and other unwanted noise. Sound monitors frequently measure sound levels exceeding 57 dB, with peaks of 82–144 dB, and even 117 dB rather continuously during medical visits and *rush hours* (15). Simply closing the incubator door is not always an option, and even then, opening and closing the doors can lead to peak noises, suddenly entering the incubator (16–18). Assuming these external sounds are a contributing factor to hearing problems or even hearing loss, one must take long-lasting cognitive damage and oversensitivity to loud (high frequency) sounds more than serious (19). Moreover, additional unwanted sound sources come from the inside of the incubator, leading to constant sources of noise pollution such as the hissing of respiratory support devices and oxygen flow. Acceptable noise levels are dependent on exposure to those levels and can have long-lasting effects on hearing even leading to hearing loss: Government regulations require over-ear protection at any time, above a level of 85 dB, a level often reached and surpassed inside of the incubator (6, 7, 15–18). On the one hand, the child is isolated and protected from outside acoustic influences, but on the other hand, she or he is exposed to medical devices inside and outside of the incubator. However, not every sound is necessarily bad for the developing infant. Studies have shown that a lack of natural voices, i.e., mother or father speaking, singing, or humming, can lead to a delayed development of speech and cognition, which in worst cases result in lasting speech deficits (7, 15, 18, 20, 21).

### Live interactive music therapy for neural development

Understanding the influence of music-based therapies and interventions on early brain development has received increasing interest over the past few decades. Neural biomarkers through DNA methylation in combination with haemodynamic measurements and near infrared spectroscopy show to be promising candidates in determining neural development in very young patients in the neonatal intensive care unit (22).

Research studying the influence of a live music-based therapeutic intervention on neural and physical development in extremely preterm born children and beyond has shown that music therapy, when administered by specially trained NICU music therapists, has beneficial results on haemodynamic markers, i.e., blood pressure and heart rate, sedation, oxygenation, and neural development as measured with near infrared spectroscopy (11, 22–25). Furthermore, the research has demonstrated that live music therapy has a much more profound effect on the wellbeing of the child than recorded music, as the music therapist can adapt to the vital signs of the child, tailoring the music-based therapeutic approach to each infant individually. This music therapeutic approach indicated a trend in the improvement of neural activation and connectivity reducing neurodevelopmental problems and improving speech and language, which provides NICU patients with better chances to participate in the society, improving their quality of life and public healthcare (22).

### Noise

Several studies have monitored and analysed sound and noise levels in the NICU, which have led noise detection to become a standard measurement (7, 20, 21). Fewer studies, however, have focused on sound and noise pollution inside of the incubator where healthcare professionals, caregivers, parents, and music therapists communicate with the infant through open incubator doors (18). Bertsch et al. have investigated noise levels generated outside and inside of an incubator and have concluded that in a no-flow respiratory support setting, the incubator protects mid and high frequency range sounds, with a strong boost of low frequencies (125 Hz) (15). Low-flow and naturally high-flow levels of the respiratory support devices mask any external sound considerable, even to the extent of eliminating every other sound. As stated, such a masking of all sounds is not only potentially harmful for speech development (masking voices, speech, and singing) but it also created a disturbing continuous sound, with parents often reporting that their children have been very restless and could not sleep, unless the vacuum cleaner or hairdryer was running after leaving the NICU.

Therefore, analysing sounds from outside of the incubator—even though these will be reduced to a minimum in the near future with alarms and other sounds being moved away from the incubator itself—as well as sounds from inside the incubator, have fuelled the current prototyping of the active noise cancellation (ANC)-incubator. As speech and singing are important markers for child development, especially cognition and speech, simple noise cancelling in the incubator does not suffice and additional measures are required.

Firstly, it is important to be able to actively cancel outside noises inside the incubator such as the sound made by the respiratory support system. Secondly, we have to be able to introduce *wanted* sounds such as speech, singing, and other music-based therapeutic interventions, without having to open the incubator doors, and unnecessarily expose the infant to peak sounds. Components of this system must take several key aspects into consideration:

1. Providing music therapy, speech, and wanted sound with closed doors;
2. active noise cancellation;
3. providing background *in utero* sounds (preferred resembling the mothers as closely as possible), mixed with speech, music-based therapeutically induced sounds, and outside world sounds, recreating the sonic environment of the womb (sonological engineering); and
4. using motion capture to monitor child and neural development (general movements, GMs) and use AI/machine learning (ML) algorithms to trigger replays of sounds, speech, and *in utero* sounds to calm the infant.

## Method

### The concept: outline, design, development, and challenges

#### Stage I: development and building of the incubator

##### Auditory range and administration, frequency, and intensity of sound

The low hearing threshold of preterm babies is around 10–15 dB at a frequency range of 500–5,000 Hz with an overall auditory range of 10–30 dB (3–11). Auditory range will be tested of each child to determine the best sound environment and level within the determined and certified maximum levels (13, 14, 16, 17, 20, 21). Frequency of administration of sound, music, and speech must be closely monitored. This may sound counter intuitive when developing a noise cancelling environment. Noise, however, while potentially damaging the neural circuitry, when replaced with a continuous stream of music therapy, speech, and other sounds, can possibly be equally as damaging (6–17). It is the human factor, administered by professionally trained NICU music therapists, nurses, and consultants, that allows the working of a high-tech medical appliance such as the ANC-incubator.

Fundamental knowledge and monitoring of human foetal development ≥28 weeks guides the development and research of the influence of clinically administered auditory stimuli including music-based therapies and interventions (22, 23). Detailed information regarding the influence of nutrition, diurnal (day/night) rhythm, maternal and familial stimuli, and cardiovascular, respiratory, intestinal, and neurological foetal development is necessary for successful development and application of the ANC-incubator. Furthermore, clinical auditory stimulation as well as action–reaction paradigms to find the right balance between under and over stimulation will have to be developed and closely evaluated, to create the perfect environment for each individual child.

##### Industrial design

The design of the ANC-incubator will be an interactive process considering the demands and wishes of the stakeholders, including neonatologists, manufactures, parents, and policymakers. Therefore, a wide range of typical industrial design-related demands and extracting techniques will be used to design and realise prototypes of the ANC-incubator. Design software, multi-physics analysis software, and 3D printing as well as moulding manufacturing techniques will be used. The interaction and visual aspects of prototypes with the stakeholders will be evaluated and developed further. Additional prototyping will be performed with existing incubators (Table 1). The prototype will be built informed by existing equipment. The Pod or Incubator Unit itself will be provided by an external manufacturer, specialised in building and developing incubators. The additions to the pod will initially be done independent from the manufacturer to allow for unbiased experimentation and measurements of the functionality of the incubator. The second stage will present the modified unit to the manufacturer to further develop and, if necessary, re-design the pod.

**Table 1:** Technological challenges.

Hardware	Software	Intersection
Motion capturing cameras	Algorithms	AI and machine learning algorithms
Microphone (for *in utero* and in the incubator)	Data retrieval and integration	Data analysis and associated decision support
dB metres		
Speakers		
Transducers		

The existing incubator will be equipped with multiple microphones and speakers to facilitate active noise cancelling inside the pod, which will be guided by software and machine learning algorithms. Additionally, we will introduce ducted systems in the air vents and oxygen supply to minimise excessive noise.

The deceptive elegance and conceptual simplicity of anti-sound is compelling; however, achieving high performance is elusive. The ANC-incubator technology generates and controls accurate anti-noise crucial to extend performance across a wider frequencies range and across a larger target zone.

Advanced active noise control algorithms enhance performance and leverage the technology convergence of edge processing, fast-loop audio processing, and acoustic transducers design.

Today's NICUs include significant exposure risk for children due to the high noise levels in the incubator cells and in NICU environments. The ANC-incubator technology covers two distinct applications identified as significant within the NICUs: ducted systems and zone-based systems. In both cases, active and passive methods combine to robustly maximise the noise reduction.

Ducted systems: These integrate patented axial arrays and algorithms within air or oxygen supply ducts (i.e., continuous positive airway pressure, CPAP) to achieve broad-band noise reduction. Low delay controllers achieve noise reduction of >20 dB over the whole hearing range to 20 kHz.

Zoned systems: Vibroacoustic noise within a sub-region or zone is minimised by using anti-sound. Novel configurations of physical and virtual sensors allow a significant increase in the control zone. The solution is effective at low to mid frequencies and is far less sensitive to the patient in the head position.

##### Monitoring system and predictive modelling

**Figure 1:**
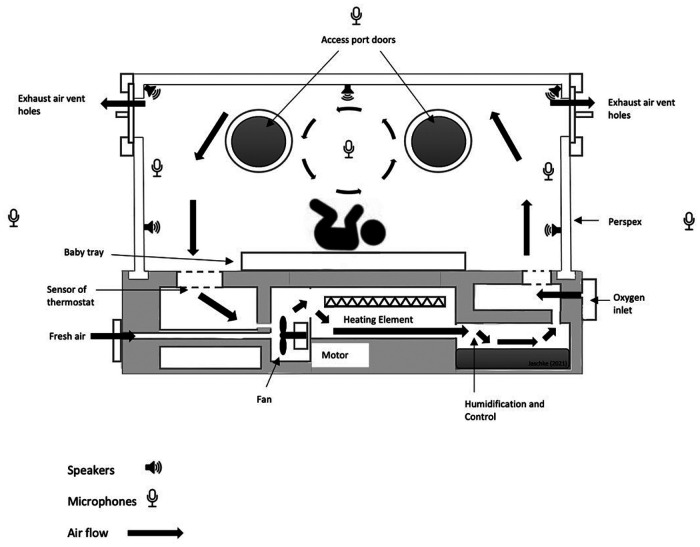
ANC-incubator sketch indicating positions of speakers and microphones to create an active noise cancelling pod. The positions of the microphones and speakers are indicative and serve for illustration only.

Several sensors suitable for non-invasive monitoring will be further developed and adjusted for foetal monitoring in the ANC-incubator. Next to motion capturing and Auditory recording (microphones and speakers) existing techniques, i.e., non-invasive, or remote monitoring of physiological signals such as electrocardiogram (ECG), breathing, oxygen saturation, and electroencephalography (EEG) will be incorporated (Figure 1). Several electrodes, ranging from contact carbon electrodes to contactless capacitive sensors, can be employed to monitor ECG and EEG of the newborn infant, in response to controlled external inputs (i.e., mothers’ recorded *in utero* sounds, parents’ voices, music-based therapeutic interventions, and general auditory stimulation). These inputs will be recorded and incorporated into both the software and machine learning algorithm to make them available when the child is in need of those sounds.

EEG measures will be performed with a clinical 8 electrode headband, which is part of the standard monitoring system in Dutch NICUs.

#### Stage II: software development for the controlled environment

##### Active noise cancelling

Analysing noise levels across the whole auditory and sonological spectrum lies at the heart of the proposed AI–machine learning paradigms in the ANC-incubator. The available multimodal recordings will be combined in a probabilistic framework enabling optimal combination of the estimated features for accurate assessment of the newborn condition and prediction of possible deterioration (7, 8, 26, 27). Feature ranking by, e.g., automatic relevance determination will be employed for identification of an optimal subset of sensors and signals to achieve accurate monitoring. The developed probabilistic models will deliver priorities derived from pathophysiological modelling of the signal sources.

Sound and especially music have been identified as a multisensory stimulus, which stimulate and interact with multiple brain areas and uniquely integrate into a vast array of neural networks, making musical sound one of the most profound triggers of neural plasticity in brain development (11, 22–25).

It is this crossroad where both interventions and natural sonological surroundings come together within a clinically implemented artificial framework. The ultimate result is to reproduce and resemble the individual sonological environment of each womb with controlled external inputs (i.e., mothers’ recorded *in utero* sounds, parents’ voices, music-based therapeutic interventions, and general auditory stimulation) (11, 22–25).

##### Human body motion capture and analysis (MoCap)

MoCap systems usually need expensive devices and technical skills that make it difficult to introduce into everyday life conditions (28–31). The recent availability of remote controllers, such as those used for gesture recognition in computer games, as well as red, green, blue plus depth (RGB-D) devices and techniques for 3D camera sensing enable us to bring automated movement analysis into everyday clinical practice without exceptional costs (28–31). We will have to investigate whether MoCap systems will still be operational with RGB-D devices during light therapy. There is the possibility of those cameras not working during light therapy, which will result in the development of both software and hardware of the cameras to adapt them to the specific NICU needs.

##### New music interaction paradigms

Musical interaction shares many characteristics with other social activities. However, commercial systems for music interaction often do not go beyond the offline transmission and storage of audio-visual information. This is partly due to the difficulties which real-time music synchronisation imposes. Recent developments in the areas of motion capture technology, brain activity sensing, music information retrieval, and machine learning have enabled the extraction of detailed interaction information that is impossible to access by direct observation or by conventional interaction technologies. By implementing AI-enhanced interaction systems, we aim to improve the efficiency of goal-driven interaction by potentiating synchronisation, entrainment, and communication of emotions among interactants; additionally, we aim to improve the understanding and accessibility of these AI systems.

##### Feedback and feedforward systems

The ANC-incubator will use both feedback and feedforward systems to cancel noise. Feedforward systems will record the noise, analyse it, and actively respond to it through single and multiple input–output controls. Additionally, AI-controlled and machine learning algorithms will use active feedforward paradigms to predict usual and expected noise, i.e., surrounding noise in the neonatal unit or the patients’ room, depending on the layout of the NICU. Feedback systems will allow the machine learning and reinforced learning algorithm to analyse and (re)interpret, adapt, and return the signal to the overall ANC-incubator system.

##### Auditory stimulation

Auditory control and stimulation will be implemented through classical, AI-based, and AI-based tracking. These approaches are based on an experimental approach and will have to be researched and tested once the prototype is built and ready.

Classical control and stimulation is based on fixed and adaptive feedforward single-input single-output active noise control using fixed acoustic sensors; fixed and adaptive feedforward multiple-input multiple-output active noise control using fixed acoustic sensors; feedforward multiple-input multiple-output active noise control using fixed acoustic sensors and uncertainty models to increase the region of silence; and feedforward multiple-input multiple-output active noise control using fixed acoustic sensors and a head motion tracking sensor to increase the region of silence.

##### AI-based control and stimulation

In the classical control approach, one uses adaptive filters, meaning that they adapt to any change of the environmental noise, even if those changes have already appeared seconds before. The AI-based control, therefore, will be investigated to assess if deep learning architectures are able to adapt to very complex signals without an assumption of linearity, and if AI-control can improve the ANC-feedback system. The AI-based system will implement deep learning for active acoustic noise control. The main idea is to employ deep learning to encode the optimal control parameters corresponding to different noises and environments. Here, all cases mentioned in classical control approach will be considered, and only the feedforward control will be substituted by ML algorithm. Furthermore, stability and performance checks can be made. In addition, reinforcement learning (RL) based control in which estimator and adaptive filter will not be used directly. Instead, primary signal will be used as feedback fed into the RL algorithm. As this method works on designing optimal control based on the numerical reward, one has to study different reward criteria and check its performance and stability.

Optimal sensor placement: as a direct by-product of the previous two algorithms, one can define the optimal sensor placement strategy.

Finally, AI-based tracking and anomaly detection will focus to track the infants’ head in real time. Here, we will investigate deep learning models for head tracking of infants, as well as recognition of facial expressions or identifications of any existing visual anomalies.

## Projected results

Incorporating different technologies into existing hardware will allow us to design a new incubator tailored to the individual needs of the child (Figure 1). A prototype is currently under construction. However, before details of the design can be implemented, the following aspects have to be researched and analysed for suitability and workability ion the NICU environment.

### Sonological engineering

Concerning sensorial influences in the NICU, a specific interrupted process weighs upon auditory brain development, which starts early in gestation.

In light of these sonological influences, it is of outmost importance to re-create the auditory environment of the womb as closely as possible. This cannot be simply done by recording the sounds and play them back on a loop, as the sonological environment inside the womb changes continuously.

Sonological engineering—the ability to record, (re)create, and administer sounds—with clinical knowledge of possible impacts on neural and behavioural development of children must follow a transdisciplinary approach, comprising and amalgamating sounds in general, structured sounds (read: music), information carrying sounds (read: language), and performance and play-back technologies (32). This combination will be dependent on (future) research with music-based therapeutic interventions in the NICU (22, 23, 33).

### Audio signal analysis

This involves the analysis and extraction of performance parameters from audio signals. The analysis will be grounded on time and spectral domain techniques, such as the Fourier transform, wavelet transform, and statistical methods, and will be based on the extraction of sound features at different semantic levels. In a lower level, we can perform a decomposition of the sound into harmonics and residual components to obtain important harmonic and residual features. Based on these lower-level features, we can compute intra-note features such as note attack duration or analysis of transients, timbre-related features, rhythmic-related features, or pitch-related features. Examples of timbre features are spectral centroid, spectral roll off, and time domain zero crossing, which give a measure of the spectral shape, the changes in spectral shape, and the noisiness of a signal. Other features, such as Mel-Frequency Cepstral Coefficients (MFCC), also based on the short-term Fourier transform, are typically used. Beat feature extraction is another important component. Regarding pitch features, we expect this to be one of the most useful feature categories and helpful for visualising deviations in intonation and pitch shifts during interactions. On a more semantic level, we can extract expressive intentions such as phrasing (measurable as tempo and dynamics), genre, or mood. One of the main challenges is to extract and visualise a set of audio parameters that are adapted to the newborns’ needs and useful for the interaction process.

A challenge is the issue of analysis of multiple sound sources, something to be approached through source separation algorithms based on convolutional neural networks.

### Motion capture technology and general movements

The use of Motion Capture in the context of music practice has been explored in a variety of artistic domains in recent years. Different modes of interaction have been suggested, from direct gestural control to a more general analysis of performer movement, to the use of software agents.

Motion Capture, therefore, will be added to monitor the neural development of the child through recording of general movements (GMs). Infants show typical as well as distinct spontaneous general movement from birth until 20 weeks post term. Infants with abnormal GMs are at a higher risk of neurological sequelae. The level of abnormality of GMs can be measured and are reflecting several neuro-pathologies. It thus allows for an early detection and therefore administration of a potential intervention.

Assessing GMs is done with infants awake lying on their back while calm and alert. No toys or pacifiers and parents should be present as this could skew the results as the infant will generally direct attention to those wanting to interact. The baby is then videoed for 5–10 min and the assessment is scored from the video. This can be a lengthy and time-consuming procedure, especially, when there are multiple measures involved over a longer period. While MoCap will not replace the human assessor, it can provide a first categorisation of the movements, speeding up the assessment process.

## Discussion

Noise levels have always been a challenging problem in neonatal intensive care units. By 23–25 weeks, all structures necessary for hearing, including the cochlea, have developed (3–5, 34). As such, most infants admitted to the NICU can hear, unless they have a congenital anomaly. From approximately 26 weeks’ gestation, foetuses and preterm infants will have the capacity to react to auditory stimuli (3–5, 11, 19–23). Sounds a foetus hears within the womb include mother's heartbeat, respiration, gait through bone conduction, and maternal voice (35). Infants show recognition to their mother's voice and, in some circumstances, their father's voice as well (35). From 30 weeks’ gestation onward, the infant can distinguish between varying speech tones and timbres and is also able to process complex auditory sounds. This point likely marks the start of speech and language development (34).

While noise levels outside of the incubator can be kept to a minimum, with external noises being moved away from the incubator itself, noise inside the incubator has been far more difficult to manage (6–23, 25). We therefore set out to prototype the ANC-incubator, amalgamating state-of-the-art active noise cancelling, knowledge from neonatology, clinical neuromusicology, and music-based therapies and interventions and the latest developments in incubator design, manufacturing, and research.

### Scientific challenges and considerations

Fundamental knowledge on foetal physiology and development is necessary, whilst also providing high-quality simulation data sets, which can be used for future research and development. Potential research and development areas include new care solutions for patients and regenerative medicine. Furthermore, solutions developed for this noise cancelling environment can be translated into solutions for current foetal predictive monitoring environments. For example, advancements in integrated photonics technology allow non-invasive measurement of concentration of compounds, opening a completely new approach for non-invasive monitoring of foetal growth and development. Fundamental knowledge on foetal development can contribute to the advances in the field of personalised regenerative medicine in a very broad sense, as it provides valuable insights into the development and formation of neuronal networks, tissues, and organs.

While active sound management and active noise cancelling have predominantly been used for the enrichment of customer experiences, translating this technology into medical applications has not yet fully been adopted. Hutchinson et al. (7) have applied active noise cancelling in their NICU to sound sources and unwanted noise from outside of the incubator. Applying this technology, they measured a decrease in unwanted sounds entering the incubator as compared to a non-ANC unit. Their elegant findings, however, have focused on noise from the outside entering the incubator, and not so much on the mechanical noise produced inside of the incubator such as respiratory systems, heating, and other sound sources. Their findings have inspired manufacturers to set out and to eliminate direct sound sources from outside of the incubator, by removing them altogether from the actual incubator unit and placing them onto wearables and rooms adjacent to the NICU. However, there are still significant noise sources inside of the incubator, i.e., CPAP or vibroacoustic noise. Both noise sources, (1) CPAP and (2) vibroacoustics, have presented major challenges in the field of noise cancellation due to their (1) close proximity to the infant (the hose is connected to a mask or nosepiece which is placed over the mouth or in the nose) and (2) constant changes to the incubator environment through adaptation of temperature, airflow, and surrounding reverberations, which will resonate with the structure of the pod. We are planning on tackling the CPAP noise through the above introduced ducted systems and the vibroacoustic noise through physical and well as digital sensors, providing a noise cancelling zone in the incubator by implementing anti-sound. These sounds can be predicted as well as measured and, thus, incorporated into the machine learning algorithm counterbalancing them in real time.

### Neuroprotection strategies

To avoid neurological complications, the ANC-incubator must ensure a stable, stress-free, physiological, neurological, and cardiovascular homeostasis. Current strategies to improve the neurological outcome are the administration of drugs that protect the brain against free radicals during episodes of hypoxia reperfusion or due to inflammation. During a time of active brain development, with a tripling of the brains surface area between 25 and 40 weeks of gestation, besides a continued neuro- and synaptogenesis, ganglions are mitigating to the thalamus, basal ganglia, and deeper cortical layers as well as development and genesis of other neurons and glial cells (36). During this period, the brain of the infant is exposed to multiple stimuli, which have a profound influence on the development of the brain at large, but also on for example heart rate, increased risk of inflammation, or hypoxia-ischemia (31). Since brain development is strongly related to many events simultaneously, one aspect can affect another. Neuroprotection strategies, therefore, must be applied across antenatal, perinatal, and postnatal factors. Different therapies are already part of the medical toolbox [for a full review, see Parikh and Juul (36)], with future directions including stem cell therapy or drug delivery and cell-specific targeting. Invasive therapies, however, still pose a risk to the infant’s short-term as well as long-term effects on neuronal development, risks that in some cases can be minimised through specific NICU Music-based therapeutic interventions (11, 23–25).

### Ethical, societal, and legal challenges and considerations

The ethical, societal, and legal aspects of this research must be identified, studied, and discussed. This includes criteria of safety and effectiveness of the ANC-incubator and clinical implications and feasibility. The potential emotional, ethical, and social implications for the prospective parents, foetuses, infants, and society as a whole are mandatory. It also includes legal and moral implications surrounding pregnancy and the start of life, e.g., the feasibility and relevance of music-based therapies and interventions as well as sonological engineering and machine learning algorithms in clinical applications. Important considerations comprise the moral relevance of maternal–foetal intertwinement and liability and classification in case of morbidity and mortality during or as a result of treatment in the ANC-incubator (although at extreme low risk), noise levels and monitoring of the audio, and possible impact of ANC on neural and physiological development (not known as of yet) as well as adhering to Medical Device Regulations.

These aspects will be considered both to ensure that the development, framing, and classification of the ANC-incubator satisfy robust ethical and legal criteria and to timely inform and discuss the implications of this device with all stakeholders. The aim is to empower co-creation between medical clinicians and researchers, non-medical researchers, caregivers, society, and patient representatives.

Over time, new differentiating features will require integration as NICU care continues to evolve. Active methods simplify integration by expanding the range of suitable active and passive noise control design trade-offs that can be optimised. This is especially applicable to the integration of AI noise control features that merge multisensory inputs. Features include noise reduction to manage child stress, improve sleep and reduce exposure, and conversely noise introduction of calming music therapy and managed carer and parental stimulation.

## Conclusion

Active noise cancelling has found its way into everyday audio applications and allows for an enriched listening experience. This technological development can be used in a multitude of ways, and especially in neonatal intensive care units, where managing sound, noise, and overstimulation has been on the clinical problem-solving agendas for decades already. Solving a complex problem like this, however, requires a multidisciplinary approach, where each discipline will realise its own shortcomings and boundaries, and in turn will allow for innovations and new avenues. Combining knowledge from active noise cancelling technology, sonological engineering, music-based therapies and interventions, medicine, and neuroscience will overcome the auditory challenges in the NICU. This allows us to create a device, which re-creates an environment as natural as possible for the neonate. By preventing auditory overstimulation and thus promoting typical neurological development, this may lead to decreasing long-term complications and morbidities post NICU, across the entire lifespan.

## Data Availability

The original contributions presented in the study are included in the article, further inquiries can be directed to the corresponding author.

## Author’s note

In our understanding, a concept and consideration paper allows for innovative, theoretical, and experimental devices, which are in development and have not yet been tested or fully constructed in the field. Therefore, the current paper introduced the idea as well as concept of an ANC-incubator amalgamating multiple disciplines to, on the one hand, develop and share the basic idea before building and testing and, on the other hand, ignite the discussion between experts to encourage collaborations and exchange of ideas, as we truly believe that the strength in developing such a device lies in de-disciplined thinking. This, in turn, will lead to identify the boundaries of each discipline and infuse the innovative aspect of building a novel device from different frontiers of knowledge.
